# Current research progress in targeted anti‐angiogenesis therapy for osteosarcoma

**DOI:** 10.1111/cpr.13102

**Published:** 2021-07-26

**Authors:** Yun Liu, Nenggan Huang, Shijie Liao, Emel Rothzerg, Felix Yao, Yihe Li, David Wood, Jiake Xu

**Affiliations:** ^1^ Department of Spine and Osteopathic Surgery First Affiliated Hospital of Guangxi Medical University Nanning China; ^2^ Research Centre for Regenerative Medicine Guangxi Key Laboratory of Regenerative Medicine Guangxi Medical University Nanning China; ^3^ Division of Regenerative Biology School of Biomedical Sciences University of Western Australia Perth WA Australia; ^4^ Department of Trauma Orthopedic and Hand Surgery First Affiliated Hospital of Guangxi Medical University Nanning China; ^5^ Perron Institute for Neurological and Translational Science OEII Medical Centre Nedlands WA Australia

**Keywords:** angiogenesis, anti‐angiogenesis, osteosarcoma, sarcoma, targeted therapy, VEGF

## Abstract

Osteosarcoma (OS) is the most common primary malignant bone tumour with a peak in incidence during adolescence. Delayed patient presentation and diagnosis is common with approximately 15% of OS patients presenting with metastatic disease at initial diagnosis. With the introduction of neoadjuvant chemotherapy in the 1970s, disease prognosis improved from 17% to 60%‐70% 5‐year survival, but outcomes have not significantly improved since then. Novel and innovative therapeutic strategies are urgently needed as an adjunct to conventional treatment modalities to improve outcomes for OS patients. Angiogenesis is crucial for tumour growth, metastasis and invasion, and its prevention will ultimately inhibit tumour growth and metastasis. Dysregulation of angiogenesis in bone microenvironment involving osteoblasts and osteoclasts might contribute to OS development. This review summarizes existing knowledge regarding pre‐clinical and developmental research of targeted anti‐angiogenic therapy for OS with the aim of highlighting the limitations associated with this application. Targeted anti‐angiogenic therapies include monoclonal antibody to VEGF (bevacizumab), tyrosine kinase inhibitors (Sorafenib, Apatinib, Pazopanib and Regorafenib) and human recombinant endostatin (Endostar). However, considering the safety and efficacy of these targeted anti‐angiogenesis therapies in clinical trials cannot be guaranteed at this point, further research is needed to completely understand and characterize targeted anti‐angiogenesis therapy in OS.

## Graphical abstract

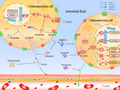

## 1 INTRODUCTION

Osteosarcoma (OS) is the most common primary malignant bone tumour. It is characterized by a high incidence of metastasis at the time of diagnosis most commonly with early haematogenous lung metastasis, a poor prognosis and a high incidence of recurrence. The use of neoadjuvant chemotherapy with multiple agents has improved the 5‐year event‐free‐survival (EFS) of non‐metastatic patients to over 70% while reducing the incidence of amputation by 10%‐20%.^1^, ^2^ However, with currently available treatment regimens, the overall 10‐year survival rate is still less than 20% in metastatic patients.^3^ Despite being known as a highly aggressive cancer, treatment for OS has remained essentially unchanged for more than 30 years, underscoring a critical need for new treatment strategies.

Compared with traditional treatment methods, targeted therapy is a type of high selectivity and low toxicity cancer treatment that uses drugs or other substances to precisely identify and attack certain types of molecules that are involved in the growth and progression of cancer (eg proteins, nucleic acid fragments and gene products).^4^ Therefore,targeted therapies are currently the focus of much anti‐cancer drug research and have been successfully applied to the treatment of chronic myeloid leukaemia, bowel, lung, breast and renal cancers.^5^, ^6^, ^7^, ^8^

In 1971, Folkman et al^9^ first described the concept that tumour growth and metastasis can be inhibited by blocking angiogenesis, establishing the basis of anti‐angiogenesis therapy. Several studies have reported the effective use of anti‐angiogenic agents such as bevacizumab in breast and cervical cancer and sorafenib in liver and thyroid cancer.^10^, ^11^ In this review, we will be discussing the molecular pathogenesis of OS, giving insight to the specific molecules which may be targeted for the effective treatment of the disease.

## 2 TUMOUR ANGIOGENESIS IN OS

Angiogenesis is a complex and highly adaptive process that is crucial for tumour growth and metastasis. The biological processes involved in angiogenesis include endothelial cell proliferation, differentiation and migration, recruitment of smooth muscle cells and maturation of blood vessels, with the processes being strictly controlled by vascular regulatory factors.^12^ Vascular regulators include both angiogenic activators such as vascular endothelial growth factors (VEGF) and platelet‐derived growth factors (PDGF), and inhibitors such as endostatin and angiostatin. An imbalance between vascular regulators will result in either angiogenesis or vascular degeneration.^13^ More than that, locally produced vascular regulatory factors in the bone microenvironment are critical to the regulation of bone homeostasis. Dysregulation of angiogenic and angiocrine activities could also lead to altered bone homeostasis, which may contribute to tumour development in bone microenvironment.^14^, ^15^, ^16^ Further, a hypoxic tumour cell environment has been reported to encourage angiogenesis in tumours by stimulating the overproduction of hypoxia inducible factor‐1 (HIF‐1) and VEGF.^17^ The activation of endothelial cells by angiogenic factors leads to the production of proteolytic enzymes which degrade the extracellular matrix. The degradation of the underlying basement membrane enables endothelial cells to proliferate and migrate to the surrounding tissue to form new vessels.^18^ These new vessels provide cancer cells with oxygen and nutrition and play an important role in cancer cell survival and metastasis. Thus, anti‐angiogenic therapies, aimed to suppress these processes, may provide an interesting approach in OS therapeutics.

Possible targets for anti‐angiogenesis therapies include transcription factors or signal pathways that directly or indirectly affect VEGF, PDGF, fibroblast growth factor (FGF), hepatocyte growth factor, integrin, cyclooxygenase (COX‐2), metalloproteinases (MMP‐2, MMP‐9) and HIF‐1.^19^, ^20^, ^21^ VEGF and its receptors (VEGFRs) are key mediators of angiogenesis in cancer.

As the most studied marker of tumour neovascularization, VEGF and VEGFRs regulate both the development of blood vessels from precursor cells during embryogenesis (vasculogenesis), and the formation of blood vessels from pre‐existing vessels (angiogenesis). It has also been shown to promote endothelium proliferation, inflammation and vascular permeability.^22^ The overexpression of VEGF is reported to be associated with disordered tumour neovascularization, destruction of endothelial cells, pericytes and basement membranes and has been implicated to promote cancer metastasis through the remodelling of microvasculature.^23^ VEGF as a therapeutic target has been validated in various types of human cancers.

**FIGURE 1:**
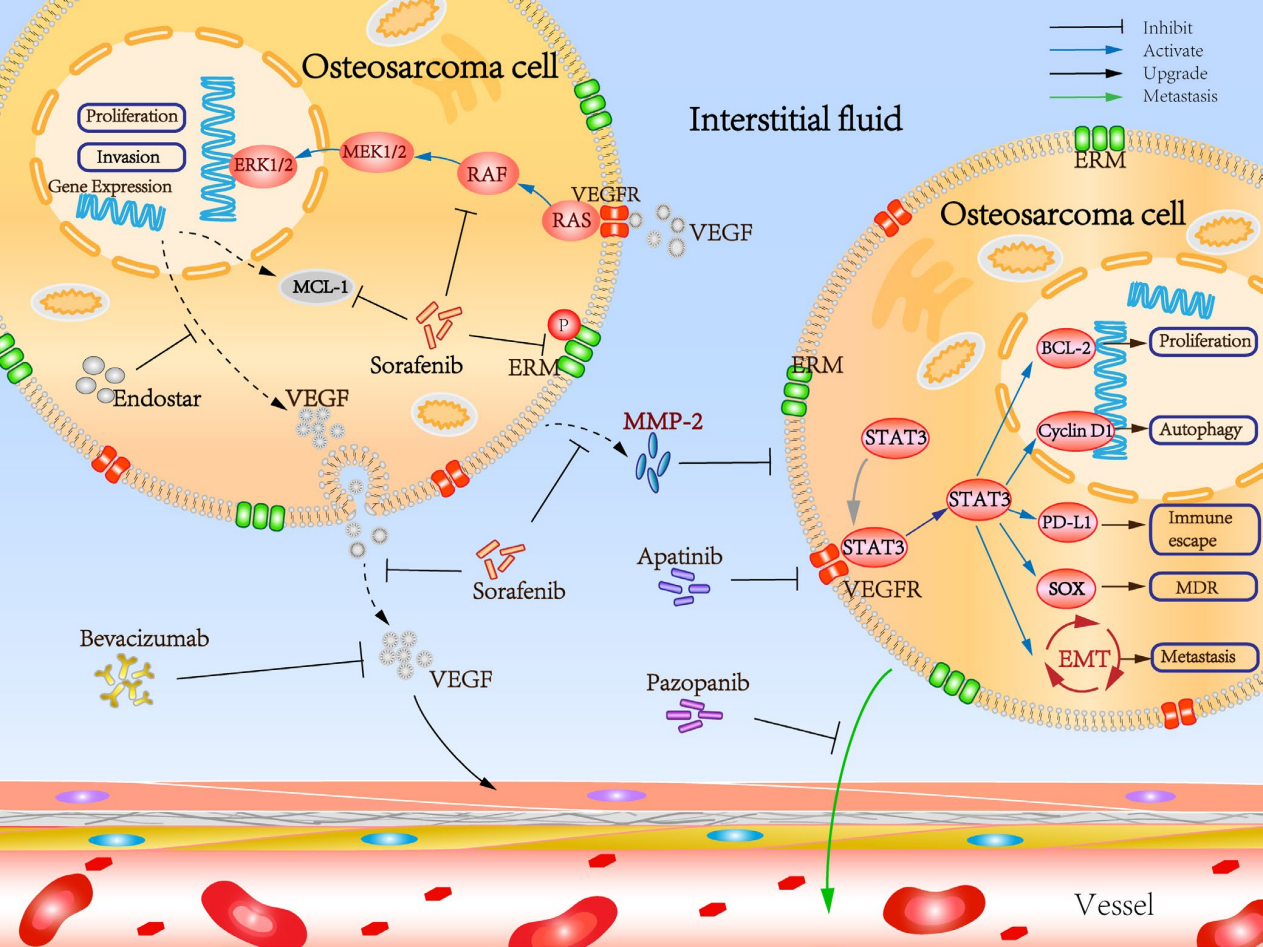
Mechanistic insights into the OS targeted anti‐angiogenesis therapy

Over‐expression of VEGF and the resultant increase in angiogenesis have been reported in a number of human cancers including OS.^24^, ^25^ VEGF levels correlate not only with the extent of tumour angiogenesis, but are a predictive measure of future metastases, and clinical prognosis.^26^ Currently, the mechanism by which the majority of anti‐angiogenic drugs prevent tumour angiogenesis is by inhibiting the VEGF or VEGF/VEGFR signalling pathway, causing tumour cells to ‘starve’ by disrupting its blood supply.^27^ In addition, anti‐angiogenic drugs may also play an anti‐cancer role in the treatment of osteosarcoma by targeting other targets (Figure 1). The utilization of such angiogenesis inhibitors has been reported to be effective in pre‐clinical trials and clinical treatments for OS (Table 1).^28^, ^29^

**TABLE 1:** Pre‐clinical studies of anti‐angiogenic inhibitors in OS

Class	Drug	In vitro or In vivo	Target	Efficacy	Refs.
Monoclonal antibody	Bevacizumab	In vivo	‐	Angiogenesis; growth↓ apoptosis↑	^40^
	Bevacizumab	In vivo	‐	Angiogenesis; growth↓	^41^
	Bevacizumab	In vivo	‐	Angiogenesis; growth↓	^42^
TKIs	Sorafenib	In vitro and In vivo	ERK1/2; MCL‐1; ezrin pathways	Proliferation; angiogenesis; metastasis↓ apoptosis↑	^48^
	Sorafenib	In vitro	VEGFR2; RET; MEK/ERK signalling pathway	Proliferation↓	^49^
	Sorafenib	In vitro	‐	Proliferation↓ apoptosis↑	^50^
	Sorafenib+everolimus	In vitro and In vivo	mTOR pathway	Proliferation; growth; metastasis; angiogenesis↓ apoptosis↑	^56^
	Apatinib	In vitro and In vivo	VEGFR2/STAT3/BCL‐2 signal pathway	Proliferation; growth↓ apoptosis; autophagy↑	^60^
	Apatinib+doxorubicin	In vitro	STAT3/Sox2 pathway	Chemoresistance↓ sensitivity to doxorubicin↑	^62^
	Pazopanib+LDM topotecan	In vitro and In vivo	‐	Angiogenesis; growth↓	^68^
	Pazopanib	In vitro and In vivo	VEGF	Metastasis↓	^22^
Endostatin	Endostar+cisplatin	In vivo	VEGF;MMP.‐9	Proliferation; angiogenesis↓	^88^
	Endostar+combretastatin A4 phosphate	In vivo	‐	Growth; angiogenesis↓	^89^
	Endostar+adriamycin	In vivo	‐	Growth; angiogenesis↓	^90^
Traditional Chinese herbal medicine	Modified Siwu decoction	In vivo	VEGF; KDR; Flt‐1	Growth; angiogenesis; metastasis↓	^94^
	Thymoquinone	In vitro and In vivo	NF‐κB; XIAP; survivin; VEGF	Growth; angiogenesis↓	^95^
	Cryptotanshinone	In vitro	VEGF	Growth; angiogenesis↓	^96^
	Triptolide	In vitro	Wnt/β‐Catenin signalling	Angiogenesis↓ Autophagy apoptosis↑	^97^

Note

↑: Up‐regulation, ↓: down‐regulation.

## 3 ANTI‐ANGIOGENIC TARGETED DRUGS FOR OS

Current management of non‐metastatic OS is multimodal consisting of aggressive chemotherapy combined with radical surgical resection. Various combinations of adjuvant chemotherapeutic agents have been used, though the majority of recent trials still use high‐dose methotrexate, doxorubicin, and cisplatin (MAP).^30^ The ‘sandwich’ therapy has reported 5‐year EFS of 60%‐70% for patients with non‐metastatic OS.^31^ However, patients with metastatic tumours that respond poorly to chemotherapy, or those with recurrent disease, still present a major clinical challenge. The survival rate for these patients still does not exceed 20% post‐therapy with established protocols.^3^, ^32^ In recent years, with a better understanding of the biology of OS growth and local environmental factors influencing disease progression, many studies have been conducted to identify possible therapeutic agents that could be of clinical use in the treatment of OS. These studies have focussed on the use of monoclonal antibody, tyrosine kinase inhibitors, recombinant human endostatin and traditional Chinese medicines. Here, we list some clinical trials of targeted anti‐angiogenic drugs for OS (Table 2).

**TABLE 2:** Clinical trials of anti‐angiogenic targeted drugs in OS

Class	Molecular targets	Therapy	Clinical trial	No. of patients	Outcome	Refs.
Monoclonal antibody	VEGF	Bevacizumab+docetaxel+ gemcitabine	Phase I	8	3/8 CR; 2/8 PR; 2/8 SD; 1/8 PD	^43^
		Bevacizumab+MAP	Phase II	31	4‐year EFS 57.5 ± 10.0%; 4‐year overall survival 83.4 ± 7.8%;	^44^
TKIs	VEGFR1‐3; RET; RAF	Sorafenib	Phase II	35	3/35 PR; 12/35 SD; 4‐month PFS 46%; m‐PFS 4 mo; overall survival 7 mo	^51^
		Sorafenib	‐	4	3/4 SD	^52^
		Sorafenib	Phase I	8	2/8 PD; 2/8 SD; 5‐year overall survival 64%	^53^
		Sorafenib+Everolimus	Phase II	38	6‐month PFS 45%	^57^
		Sorafenib+denosumab	‐	1	Complete metabolic remission	^58^
	VEGFR1‐2; Kit; c‐SRC; RET	Apatinib	‐	1	1/1 PR	^63^
		Apatinib	Phase I	10	2/10 PR; 5/10 SD; ORR 20.0%; DCR 70.0%; 6‐month PFS 60%; median overall survival 14 mo; m‐PFS 7.5 mo	^64^
		Apatinib	Phase II	37	16/37 PR; 4‐month PFS 57%; m‐PFS 4.5 mo; median overall survival 9.87 mo	^65^
		Apatinib	Phase II	27	ORR 25.93%; DCR 66.67%; m‐PFS 3.5 mo; median overall survival 9.5 mo	^66^
	VEGFR; PDGFR‐a/3; c‐Kit	Pazopanib	‐	3	3/3 CR	^69^
		Pazopanib	‐	3	3/3 PR	^70^
	VEGFR1‐3; BRAF; KIT; PDGFRB; RAF; RET; AKT; ERK	Regorafenib	Phase I	3	1/3 PR	^79^
		Regorafenib	Phase II	26	DCR 64%; PR 8%; m‐PFS 16.4 wk	^80^
		Regorafenib	Phase II	42	m‐PFS 3.6 mo; 3/22 PR	^81^
Endostatin	VEGF	Endostar+chemotherapy	Phase II	166	5‐year EFS 70%	^91^
		Endostar+chemotherapy	Phase II	388	5‐year overall survival 87%; 5‐year DMFS 79%	^92^
		Endostar+chemotherapy	‐	1	PR	^93^

Abbreviation: EFS, event‐free survival; PFS, progression‐free survival; m‐PFS, median progression‐free survival; SD, stable disease; PR, partial response; ORR, objective response rate (complete response +partial response); CR, clinical response; DCR, disease control rate; DMFS, distant metastasis‐free survival.

### 3.1 Monoclonal antibody

Monoclonal antibodies (mAb) are laboratory engineered proteins which serve as substitute antibodies that can restore, enhance or mimic the immune system's attack on specific target cancer cells. mAb targeted drugs, which recognize and bind specific receptors or associated antigens on the surface of tumours, have been shown to improve the efficacy and reduce the toxic side effects of drugs in normal tissues.^33^, ^34^ mAbs targeting VEGF was shown to drastically suppress tumour growth in vivo. The recombinant human VEGF mAb bevacizumab was approved for clinical use by the US Food and Drug Administration (FDA) in 2004 and is the first VEGF inhibitor to be approved for use in the treatment of cancer.^35^ It is a single‐target anti‐VEGF drug that binds to soluble VEGF, preventing receptor binding and inhibiting endothelial cell proliferation and vessel formation. It has been reported to be beneficial in solid tumours such as colorectal cancer, lung cancer, renal cell carcinoma and prostate cancer.^36^, ^37^, ^38^, ^39^

Several pre‐clinical OS studies have confirmed the anti‐angiogenesis and anti‐cancer effects of bevacizumab in OS cell lines. Zhong ZY et al^40^ reported that bevacizumab significantly inhibited angiogenesis in OS, leading to cell apoptosis and a reduction in tumour size. The reported inhibition rate of bevacizumab on tumours is 32.87%, and this effect was synergistic with radiotherapy. Similarly, Zhao et al^41^ reported that bevacizumab had strong anti‐angiogenic activity in a population of OS simulated nude mice strain, resulting in inhibition of tumour growth. Valery et al^42^ also reported the effective application of bavacizumab in the treatment of transplanted canine OS in mice. The authors suggested that the growth rate and microvascular density change observed in transplanted canine OS cells had a dose‐dependent positive correlation with bevacizumab. This novel finding demonstrates its possible application as an effective neoadjuvant therapeutic agent for OS patients.

Many distinct escape mechanisms have been shown to protect malignant cells from immune recognition and destruction in the tumour microenvironment, and these mechanisms make the response from single‐target drugs like bevacizumab short‐lived. As a result, it is often used in combination with other chemotherapeutic drugs in clinical trials. In a single‐centre trial using docetaxel, bevacizumab and gemcitabine for the treatment of high‐risk sarcomas with metastasis and/or recurrence in adolescents/young adults, Kuo et al^43^ reported that, among 8 patients with OS, only 3 achieved clinical remission, while 2 patients achieved partial response (PR), 2 patients had a stable disease (SD), and 1 patient had a progressive disease. The tumour control rate was 88% (7/8), demonstrating the potential effectiveness of combining bevacizumab with other anti‐tumour agents in the treatment of young patients with high‐risk sarcomas. Recently, a phase II clinical trial by Navid et al^44^ reported a significant improvement in 4‐year EFS and total survival rates among patients with limited resectable OS treated with bevacizumab+MAP. From a total of 31 patients with localized OS, the EFS and total survival rates were 57.5 ± 10.0% and 83.4 ± 7.8%, respectively, and 8 (28%) of 29 evaluable patients had a histological response to preoperative chemotherapy. Of these patients, the addition of bevacizumab did not improve histological response, a strong predictor of outcome in OS, which correlated to no apparent improvement in EFS or overall survival.^44^ Authors also reported that approximately half of the study cohort experienced a reduction in wound healing which is a complex process that includes a vascularization phase similar to that supports tumour growth with VEGF and VEGFRs as key regulators.

Bevacizumab has been shown to normalize tumour vasculature both in the laboratory and in patients with other types of cancers and therefore improve access for chemotherapy. In combination with other chemotherapeutic agents, it has been shown to be efficacious in the treatment of several cancers clinically by increasing tumour response and prognosis. However, there is a need for further investigation to the use of bevcizumab alone or in combination with other agents, to demonstrate its efficacy in OS patients.

### 3.2 Anti‐angiogenic tyrosine kinase inhibitors (TKIs)

Tyrosine kinases are important mediators of the signalling cascade.^45^ It can be classified as either a receptor tyrosine kinase (RTKs) or non‐receptor tyrosine kinase (NRTKs). The RTK family includes INSR and many growth factors (such as VEGF, FGF, PDGF and EGF) receptors.^46^ TKIs may be single‐ or multi‐target inhibitors which have advantages and disadvantages related to pharmacokinetics, selectivity, potential resistance mechanisms and tumour environment. Since most tumours display multiple signalling pathways, many of the inhibitors in clinical development are multi‐targeted to affect a wide range of targeted kinases increasing their efficacy and are less susceptible to the development of drug resistance. Recently, TKIs have received increased attention with major breakthroughs in their clinical applications, bringing hope to oncologists and cancer patients.^13^ Clinicians have attempted to apply TKIs for the development of targeted chemotherapies for OS, and some successes have been achieved for some of the agents, including sorafenib, apatinib, pazopanib and regorafenib, which have been reported to be effective in clinical trials. These results support the hypothesis that specific TKIs may be effective to regulate tumorigenesis and/or metastasis in OS.

#### 3.2.1 Sorafenib

Sorafenib is the first oral multi‐kinase inhibitor that targets VEGFR‐1, VEGFR‐2 and VEGFR‐3 to inhibit angiogenesis, the RET gene also known as RET/PTC rearrangement, RAF (including BRAFV600E) and platelet‐derived growth factor receptors to inhibit tumour progression.^47^ Its use for the treatment of advanced renal cell carcinoma, hepatocellular carcinoma, acute myeloid leukaemia and thyroid carcinoma has been approved by the FDA. In a pre‐clinical study, Ymera et al^48^ reported that sorafenib inhibited the ERK1/2, MCL‐1 and ERM signal pathways, and the proliferation of OS cells in a dose‐dependent manner. It also induced apoptosis and inhibited the formation of new blood vessels. Mei et al^49^ reported that sorafenib inhibits the proliferation of OS cells by influencing the VEGFR2, RET and MEK/ERK signalling pathways. In addition, Wolfesberger et al^50^ reported that sorafenib showed potent anti‐tumour activity against canine OS cells in vitro, suggesting its therapeutic potential as a tool in the management of bone cancer in dogs.

Three studies to date have evaluated the clinical efficacy of sorafenib alone for the treatment of OS. A phase II trial of sorafenib by the Italian Sarcoma Group in 2012^51^ reported that of 35 patients aged >14 years enrolled with recurrent/unresectable tumours who received sorafenib, progression‐free survival at 4 months was 46%. Although some unprecedented long‐lasting responses were achieved, for the majority its effect was short‐lived with only 29% of patients reported to have stabilized progression at 6 months. In another trial, 3 out of 4 patients with relapsed OS post‐chemotherapy who obtained treatment with sorafenib had SD with a median response time of 3 months, after which their condition deteriorated.^52^ Furthermore, a study done in Poland by Raciborska et al^53^ reported that among 8 patients with refractory OS with a median age of 18years, treatment with sorafenib achieved remission in 6 patients with an overall response rate of 75%. There were no serious toxicities, and sorafenib was well tolerated in young patients with bone tumours. In summary, these trials show that sorafenib was able to temporarily inhibit OS progression, although the benefit was minimal and short‐lived. Therapy with sorafenib alone demonstrated temporary tumour stabilization in OS, indicating that more effective combination treatment regimens are required to achieve permanent remission.

Oncogenic activation of mTOR signalling significantly contributes to the progression of different types of cancers including OS, and the PI3K/Akt/mTOR pathway has been implicated in the metastatic behaviour of OS.^54^, ^55^ In 2013, a pre‐clinical study by Pignochino et al^56^ confirmed that the lack of efficacy of sorafenib as a single agent has been attributed to its action on the mTOR pathway, so the coadministration of sorafenib with an mTOR inhibitor may improve its effectiveness. Subsequently, Pignochino et al^57^ conducted a non‐randomized phase 2 clinical trial. They achieved a 10% overall response rate when treating adults with advanced recurrent/unresectable disease with combination treatment of sorafenib and everolimus, though the benefits were unsustained and did not attain the prespecified target of 6‐month progression‐free survival (PFS) of 50% or greater.

Cathomas et al^58^ looked at the role of receptor activator of nuclear factor‐kB ligand (RANKL) inhibitors in addition to sorafenib as a combination treatment option for unresectable OS after chemotherapy. The authors reported complete sustained metabolic remission ongoing for over 18 months following treatment with sorafenib and denosumab. This finding confirms pre‐clinical data on RANK/RANKL inhibition in OS, but larger clinical trials are required to determine its efficacy in this patient population.

Although sorafenib is a relatively new drug, it is now considered first‐line treatment for advanced hepatocellular carcinoma and renal cell carcinoma. Its effect on OS has yet been studied on a large scale, and more clinical data are needed to properly assess its efficacy as a possible treatment option particularly for patients with metastatic disease and refractory OS. Current studies have demonstrated that the effect of sorafenib as a monotherapy for OS patients was unsustainable. Different pathways involved in the pathogenesis of OS warrant further investigation looking at multiple‐targeted therapeutic approaches which may yield major improvements in the management of the disease.

#### 3.2.2 Apatinib

Apatinib is a small molecule tyrosine kinase inhibitor that highly and selectively targets VEGFR‐2 and also inhibits the activities of VEGFR‐1, Kit, c‐SRC and RET,^59^ leading to the inhibition of endothelial cell migration and proliferation, and decrease in tumour microvascular density. Compared with sorafenib, its effects on VEGF mediated endothelial cell migration and proliferation are more potent. It is also considerably cheaper as an alternative anti‐tumour agent. In the related study of OS, Liu et al^60^ reported that apatinib induced autophagy and apoptosis in OS by the inactivation of the VEGFR2/STAT3/BCL‐2 signalling pathway. When combined with autophagy inhibitors, its anti‐tumour effects were further enhanced. Signal transducers and activators of transcription 3 (STAT3) has been implicated as an oncogene and therapeutic target in a variety of neoplastic diseases including OS.^61^ In line with this, a recent study reported that apatinib inhibits SRY‐Box Transcription Factor 2 (Sox2) via the STAT3 signalling pathway, significantly reducing the doxorubicin‐induced stem phenotype and metastasis of OS cells and providing a new therapeutic strategy for overcoming adriamycin‐induced drug resistance in the treatment of OS.^62^ Unfortunately, the anti‐angiogenic effect of apatinib has not been demonstrated in pre‐clinical studies of OS.

Apatinib is reported to be safe and efficacious to use in patients with advanced OS. Clinical trials by Zhou et al^63^ reported that apatinib showed promising efficacy and an acceptable safety profile in a trial of patients with metastatic OS. In this study, a patient with pulmonary metastases achieved PR 11 months post‐treatment without any serious drug‐related side effects. Zheng et al^64^ suggested that apatinib treatment as a single agent in 10 patients with advanced OS with pulmonary metastases, whose first‐line chemotherapy had failed, resulted in a 60% PFS and 70.0% disease control rate (DCR) at six months. The median PFS was 7.5 months and median overall survival was 14 months, indicating that apatinib is an effective single‐drug treatment option for patients with OS and associated pulmonary metastases, though randomized controlled trials based on this data are required to further evaluate apatinib activity. Phase II clinical trial of 37 patients with progressive relapsed or unresectable OS by Lu et al^65^ treated with apatinib found an objective response rate (ORR) of 43.24% (16/37) and a 4‐month PFS rate of 56.76% (95% CI, 39.43%‐70.84%). The authors also reported a median progression‐free survival (m‐PFS) and total survival rate of 4.50 months (95% CI, 3.47‐6.27) and 9.87 months (95% CI, 7.97‐18.93), respectively, confirming its high objective efficacy and low toxicity. Another review of 27 OS cases treated with an initial apatinib dose of 500 mg/qd by Tian et al^66^ found an ORR of 25.93% and DCR of 66.67%. This study reported a median progression‐free survival of 3.5 months (95% CI, 2.5‐4.8 months), and median overall survival of 9.5 months (95% CI, 7.8‐10.5 months).

For patients with advanced OS non‐responsive to chemotherapy, apatinib proved to be effective in prolonging PFS in previous multi‐centre trials and was consequently added to the National Comprehensive Cancer Network guidelines in the United States as a second‐line therapy. However, it must be noted that these promising results have been limited to mostly retrospective studies with small population sizes without controls. Additional studies are needed to fully calculate the efficacy of apatinib for the treatment of OS.

#### 3.2.3 Pazopanib

Pazopanib, a second‐generation multi‐target TKI, is approved for the treatment of soft tissue sarcomas and has a high affinity for VEGF receptors including PDGFR‐a/3 and c‐Kit receptors.^67^ Sushil et al^68^ reported that pazopanib exerts its anti‐cancer effect through the inhibition of both angiogenic and oncogenic signal pathways in OS mouse models. The anti‐angiogenic effects of pazopanib were significantly enhanced when combined with the chemotherapy drug Topotecan. Tanaka et al^22^ showed that daily administration of pazopanib given to C3H mice bearing LM8 OS xenografts reduced the rate and size of pulmonary metastasis. Pazopanib inhibited the destruction of vascular barriers preventing trans‐endothelial migration of tumour cells, leading to a reduced incidence of pulmonary metastases in vivo.

A few studies have suggested that the use of pazopanib as an inhibitor of these tyrosine kinases can lead to an improved clinical response. A study by Danish researchers^69^ reported PR in 3 patients with advanced osteogenic sarcoma following treatment with pazopanib. Umeda et al^70^ described 3 patients with recurrent OS who survived for more than 21 months after treatment with pazopanib. There are also ongoing clinical trials and unreported completed trials looking at pazopanib as a viable therapeutic option for OS patients.^71^ So far, these studies have shown that pazopanib exhibited favourable clinical benefit and a tolerable toxicity profile compared to other TKIs.

These reports indicate that pazopanib has strong anti‐tumour activity and may contribute to improved survival. The response rates, PFS and overall survival are comparable with the efficacy shown using sorafenib alone or in combination with everolimus. The toxicity profile of pazopanib also appeared to be acceptable for treatment. Recently, paxopanib was approved for second‐line treatment in non‐adiopocytic soft tissue sarcoma (STS) after failure of standard chemotherapy based on the PALETTE study—a double‐blind, randomized, phase 3 trial which was conducted globally comparing the efficacy of pazopanib versus placebo for the treatment of metastatic STS.^72^, ^73^, ^74^ Despite data suggesting that pazopanib can stabilize disease, the PFS and overall survival are was still poor for patients with metastatic OS. Future studies on pazopanib alone or in combination with chemotherapy and/or radiation therapy could be investigated in a randomized trial.

#### 3.2.4 Regorafenib

Regorafenib is a small molecule multi‐kinase inhibitor, which inhibits the activity of VEGFR1‐3, KIT, PDGFRB, RAF, RET and BRAF, and has been demonstrated to increase the overall survival of patients with US FDA approval for use in metastatic colorectal cancer, advanced gastrointestinal stromal tumours and hepatocellular carcinoma.^75^, ^76^ It has also been theorized to inhibit the progression of OS by inhibiting AKT and ERK‐mediated signalling pathways. Its biochemical characteristics are similar to sorafenib, though its pharmacological effects are noticeably stronger.^77^, ^78^ Regorafenib has shown some significant results in clinical trials of OS. However, the anti‐angiogenesis mechanism in the disease has not yet been illustrated.

Phase I clinical trials conducted by Klaus et al^79^ showed that one third of patients with advanced OS achieved PR with regorafenib treatment, providing a preliminary evidence of its safety and anti‐tumour capabilities. Subsequent non‐comparative, double‐blind, placebo‐controlled, phase 2 trial done by Duffaud et al^80^ found that regorafenib was well tolerated. The regorafenib group also achieved a median PFS of 16.4 weeks, a DCR 65% and a PR in 8% of patients. A second randomized, double‐blinded, phase II study^81^ reported that regorafenib improved the progression time of adult progressive metastatic OS compared with placebo (m‐PFS of 3.6 months and 1.7 months, respectively). The authors concluded that regorafenib demonstrated clinically meaningful anti‐tumour activity in adult patients with recurrent, progressive, metastatic OS after failure of conventional chemotherapy, with a positive effect on delaying disease progression. It is possible that regorafenib may have an important role as a therapeutic agent complementary to the standard regimens used against OS.

### 3.3 Endostatin

Endostatin, isolated from a culture medium of rat endothelioma by O’Reilly MS et al in 1997,^82^ is an endogenous protein which is a potent inhibitor of VEGF expression and angiogenesis.^83^ In 2005, Endostar (ES) or human recombinant endostatin was developed independently and first used therapeutically for lung, nasopharyngeal and malignant tumours of the digestive system.^84^, ^85^, ^86^ Recombinant human endostatin is considered safe for clinical use and displays multi‐target tumour suppression potential through the inhibition of VEGF expression, and the activation of ERK, AKT and MAPK pathways.^87^ Recent studies demonstrated the clinical benefit of ES as an anti‐angiogenesis therapeutic agent for OS when used in combination with other chemotherapy agents.

Pre‐clinical studies reported that the combination of ES and chemotherapy agents significantly inhibited tumour angiogenesis and growth. Zhang XL et al^88^ reported that the combination of ES and cisplatin inhibited the growth of OS via the inhibition of VEGF and MMP‐9 expression. Fu et al^89^ reported that the combination of the anti‐angiogenesis agent ES and the vascular disrupting agents (VDA), combretastatin A4 phosphate (CA4P), had a good anti‐tumour and anti‐angiogenesis effect in OS mice models, with no significant toxicity. Synergistic anti‐tumour effects of ES with doxorubicin and Adriamycin have also been reported in OS mice models.^90^

The anti‐tumour activity of ES has been demonstrated in clinical trials. Xu et al^91^ evaluated the clinical efficacy of ES combined with chemotherapy in the treatment of OS. In a group of 116 newly diagnosed patients with OS enrolled to receive chemotherapy with or without ES, there was no significant difference in the histological response between the ES treatment and non‐treatment groups. However, ES treatment significantly inhibited the expression of VEGF and presence of micro‐vessels induced by chemotherapy. ES treatment did not affect the overall survival rate, but increased the EFS rate and reduced the occurrence of metastases. A recent non‐randomized controlled trial reported that 388 patients with non‐metastatic conventional OS, who were treated with ES combined with chemotherapy, achieved significantly increased 5‐year metastasis‐free survival rate of 79% (versus 61% in the control group) and a 5‐year survival rate of 87% (versus 74% in the control group).^92^ Moreover, a study by Jiang et al^93^ reported that a paediatric OS patient with pulmonary metastasis and malignant pleural effusion, who was treated with surgical resection combined with ES and chemotherapy, obtained pathologic complete remission and was in PFS. The experiences above indicate that ES combined with chemotherapy had significant activity to increase survival rates in patients with advanced sarcomas, with tolerable side effects, and warrant further investigation for future therapeutic regimens.

### 3.4 Traditional Chinese herbal medicine

In recent years, the synergistic effects of traditional Chinese herbal medicine (CHM) combined with radio‐ and chemotherapy have been gaining increasing significance as its effectiveness as an anti‐angiogenetic agent are being explored. Huang YM et al^94^ showed that the modified Siwu decoction, a traditional Chinese medical formula, prevents the growth and metastasis of malignant bone tumour in vivo via the inhibition of VEGF, KDR, Flt‐1 expression and angiogenesis in a dose‐dependent manner. Peng et al^95^ reported that thymoquinone, an extract of black fennel, could inhibit tumour angiogenesis and growth via the inhibition of NF‐κB and its regulatory molecules. Xu YM et al^96^ also reported that the fat‐soluble effective monomer cryptotanshinone, derived from the plant salvia miltiorrhiza (also known as red sage), can effectively reduce the level of VEGF expression in OS cells, thus inhibiting angiogenesis. Moreover, a recent study by Li et al^97^ reported that triptolide, the active natural product isolated from the medicinal plant Tripterygium wilfordii, can inhibit angiogenesis and induce apoptosis of OS cells by inhibiting the expression of HIF‐1 and VEGF via the Wnt/catenin signalling pathway. Collectively, these studies demonstrate that traditional Chinese medicines have the potential to become an adjunct treatment for OS and other malignancies in combination with conventional regimens by promoting anti‐angiogenesis effects. However, the majority of these studies have only been conducted in vitro, and significant further investigative studies are required to determine its efficacy in vivo.

## 4 SUMMARY AND FUTURE PROSPECTS

There is no established systemic treatment option for advanced or unresectable OS progressing after standard chemotherapy, and as such, the survival rate of patients with OS has not improved significantly in recent years. Angiogenesis is a key factor affecting tumour growth and metastasis, and theoretically, anti‐angiogenic therapeutic agents present potentially novel therapies for various cancer types.

Recently, progress has been made in the development and application of targeted anti‐angiogenic drugs, providing much‐needed relief in the search of therapeutic alternatives for OS patients. Much of this research has focussed on cases of refractory and/or metastatic disease following failed traditional chemotherapy and surgical resection. However, the search for alternative targeted anti‐angiogenic regimens is still in its infancy and a long way from achieving desired clinical applications. New research has generated plenty of excitement, but still faces a myriad of questions before it can be widely adopted for clinical use. For instance, how do we predict the therapeutic effect of an anti‐angiogenetic targeted therapeutic agent for OS, and which combination of new agent with traditional therapies would be most effective. More detailed clinical studies are needed to establish reasonable norms and guidelines for the application of these plausible therapeutic alternatives. With much‐needed technological developments and extensive research, targeted anti‐angiogenesis therapy could become a potent and effective weapon in our fight to effectively manage patients with OS.

## CONFLICT OF INTEREST

All authors declare that they have no conflict of interest.

## AUTHOR CONTRIBUTIONS

Yun Liu and Nenggan Huang conducted research and drafted the manuscript. Shijie Liao, Emel Rothzerg, Yihe Li and Felix Yao provided assistance in the process of revised drafting manuscript and figure and tables construction. David Wood and Jiake Xu contributed to conceptual framework, supervised the study and revised the manuscript.

## Data Availability

The data that support the findings of this study are available from the corresponding author upon reasonable request.
